# Pseudohypercreatininemia after surgery for aortic dissection: a case report

**DOI:** 10.1186/s12882-023-03275-2

**Published:** 2023-07-25

**Authors:** Ayako Tasaki, Makoto Fukuda, Yuki Ikeda, Masatora Yamasaki, Ikko Yamaguchi, Shinichi Aishima, Motoaki Miyazono

**Affiliations:** 1 Department of Internal Medicine, Division of Nephrology, Faculty of Medicine, Saga University, Saga, 849-0937 Japan; 2 Department of Clinical Laboratory Medicine, Faculty of Medicine, Saga University, Saga, Japan; 3 Department of Internal Medicine, Division of Pathology, Faculty of Medicine, Saga University, Saga, Japan

**Keywords:** Pseudohypercreatininemia, Acute kidney injury, Enzymatic method, Case report, IgG4

## Abstract

**Background:**

Elevated creatinine concentrations often indicate acute renal injury and renal biopsies are considered in this situation. However,pseudohypercreatininemia is potential cause of elevated creatinine concentrations, and invasive interventions should be avoided.

**Case presentation:**

A 54-year-old woman underwent surgery for descending aortic dissection.Nine days postoperatively, her creatinine concentration increased from 1 mg/dl to 5.78 mg/dl (normal range, 0.47–0.7 mg/dl). Azotemia and hyperkalemia were absent and physical examination findings were unremarkable. Cystatin C concentration was 1.56 mg/l (normal range, 0.56–0.8 mg/l) and pseudohypercreatininemia was suspected. Testing with different reagents showed a creatinine concentration of 0.84 mg/dl. Immunoglobulin (Ig)G was markedly elevated, and creatinine and IgG fluctuated in parallel, suggesting the cause of the pseudohypercreatininemia. IgG4 was also elevated at 844 mg/dl. Immunosuppressive steroid therapy effectively decreased the IgG concentration and resolved the pseudohypercreatininemia.

**Conclusions:**

In cases of elevated creatinine concentration with the presence of abnormal proteins, pseudohypercreatininemia should be considered. We report a rare case of pseudohypercreatininemia caused by polyclonal IgG.

## Background

Creatinine may be measured using the Jaffe method and enzymatic methods [1]. The latter has high specificity and is used by many medical institutions but is affected by paraproteins and some drugs. There are reports of pseudohypercreatininemia caused by immunoglobulin (Ig)M [2, 3], but to the best of our knowledge, there are no reports of pseudohypercreatininemia caused by IgG. We report a case of pseudohypercreatininemia caused by polyclonal IgG interference in an enzymatic test routinely performed for creatinine measurement.

## Case presentation

**Fig. 1:**
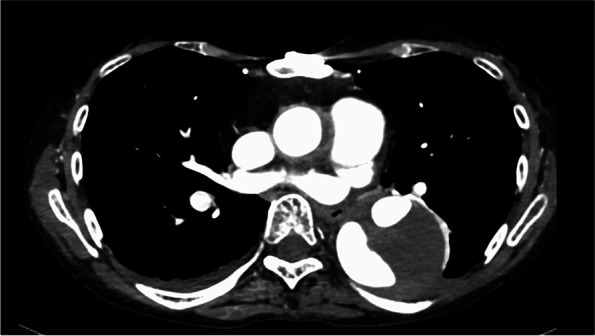
Computed tomography image 9 days before surgery

A 54-year-old woman underwent surgery for acute aortic dissection, Stanford type A, 4 years previously. She also underwent stent graft insertion for a dissecting abdominal aortic aneurysm 3 years previously. At the current admission, she had developed another aortic dissection in the descending aorta (Fig. 1) and underwent graft replacement on X date. She had multiple aortic dissections at a young age, but had no findings or family history of Marfan syndrome or Ehlers–Danlos syndrome. Because of severe intraoperative bleeding, she was transfused with 10 units of red blood cells, 4 units of fresh-frozen plasma, and 10 units of platelets. She remained in the intensive care unit for 6 days postoperatively, and her creatinine concentration was approximately 1 mg/dl. However, on postoperative day 9, her creatinine concentration increased to 5.78 mg/dl. Urinalysis showed occult blood and proteinuria (Table 1); therefore, we considered performing a renal biopsy.

**Table 1:** Urine and blood test results on postoperative day 13

Urine tests	Value	Normal Value	Units
Protein	0.89	< 0.15	g per 24 h
Erythrocytes	27.7	0–4	/HPF
Leukocytes	35.3	0–4	/HPF
Nitrites	positive	negative	
Blood tests			
Leukocytes	8900	3,000–7,800	/μl
Erythrocyte	3,050,000	3,530,000–4660000	/μl
Hemoglobin	9.4	10.6–14.4	g/dl
Platelets	2,500,000	138,000–309000	/μl
Total protein	6.8	6.5–8.0	g/dl
Albumin	2.7	4.0–5.2	g/dl
Blood urea nitrogen	10.2	7–24	mg/dl
Creatinine	7.68	0.47–0.7	mg/dl
Cystatin C	1.49	0.56–0.8	mg/l
eGFR	4.9	≧60	ml/min/1.73 m^2^
eGFRcys	43.6	≧60	ml/min/1.73 m^2^
Sodium	135	136–145	mmol/l
Potassium	4.5	3.3–4.8	mmol/l
Chloride	97	98–110	mmol/l
C-reactive protein	2.72	0–0.3	mg/dl
Immunoglobulin G	3165	870–1700	mg/dl
Immunoglobulin A	350	110–410	mg/dl
Immunoglobulin M	65	46–260	mg/dl
ANA	negative	negative	
Cryoglobulin	negative	negative	
PR3-ANCA	negative	negative	
MPO-ANCA	negative	negative	
GBM-Ab	negative	negative	

*eGFR* Estimated glomerular filtration rate, *eGFRcys* eGFR with cystatin, *C ANA* Antinuclear antibody, *PR3-ANCA* Proteinase 3-antineutrophil cytoplasmic antibody, *MPO-ANCA* Myeloperoxidase-antineutrophil cytoplasmic antibody, *GBM-Ab* glomerular basement membrane antibody

However, because there was no evidence of azotemia or hyperkalemia, pseudohypercreatininemia was considered as a differential diagnosis. She had no uremic symptoms, such as general malaise or decreased appetite, and her urinary status was good. Her cystatin C concentration measured with creatinine was 1.56 mg/l (normal range, 0.56–0.8 mg/l), which supported the presence of pseudohypercreatininemia. Creatinine was measured by a different enzymatic method and the result was 0.84 mg/dl. Inulin clearance was 41 ml/min, which was consistent with pseudohypercreatininemia (Table 2).

**Table 2:** Postoperative cystatin C concentrations, and creatinine concentrations using different measurement methods

	Normal value	POD 7	POD 9	POD 13	POD 83
Creatinine (mg/dl)	0.47–0.7				
Enzymatic method (*Shigunasuoto*)		1	5.78	7.68	6.6
Enzymatic method (*Detamina-L*)		0.95	0.84	0.75	1.34
High-performance liquid chromatography			0.71		0.54
Cystatin C (mg/dl)	0.56–0.8	1.8	1.56	1.49	1.34

*POD* Postoperative day

**Fig. 2:**
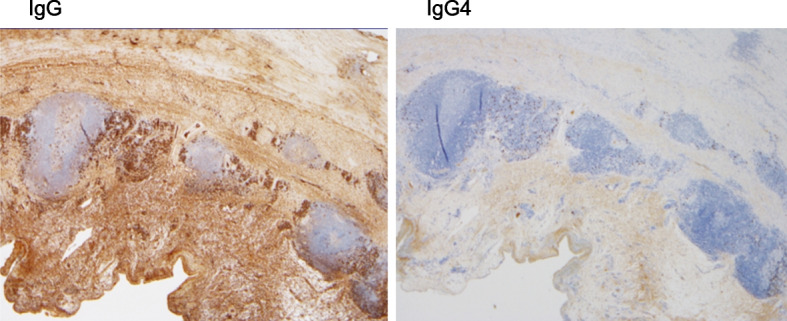
Pathological images. Left image: staining for IgG; right image: staining for IgG4 (magnification in both images: × 20). IgG, immunoglobulin G

**Fig. 3:**
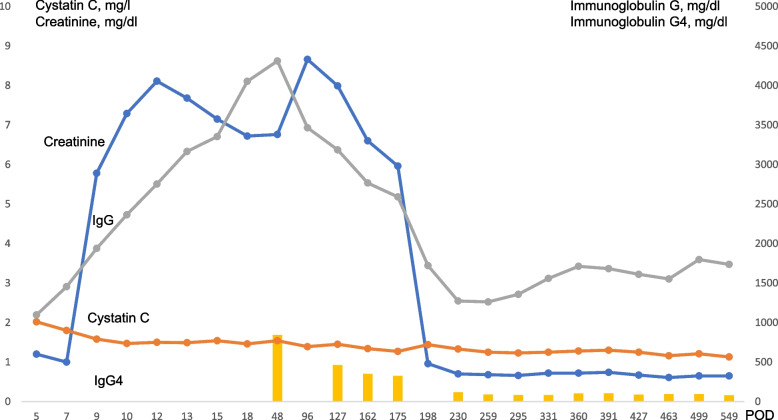
The patient’s clinical course. IgG, immunoglobulin G; POD, postoperative day

The patient had an abnormal total protein/albumin ratio, suggesting the presence of abnormal proteins. Electrophoresis did not reveal any distinct M-proteins; however, IgG was markedly elevated, concurrent with the elevated creatinine concentration, and the IgG concentration fluctuated over time in parallel with the creatinine concentration. Therefore, paraproteins were considered a factor influencing the creatinine reagents. We measured IgG4 and found a very high concentration at 844 mg/dl, suggesting IgG4-related disease. However, computed tomography and gallium scintigraphy showed no obvious mass lesions other than pancreatic cysts. Histopathology of the resected aorta showed lymphatic follicles and plasma cell infiltration from the aortic adventitia to the adipose tissue, and IgG4-positive cell infiltration was observed in some areas. However, the IgG4/IgG ratio was approximately 15%, which did not fulfill the diagnostic criteria for IgG4-related inflammatory abdominal aneurysm or IgG4-related periarteritis (Fig. 2). IgG4 and creatinine concentrations changed in tandem and were considered to be the cause of the pseudohypercreatininemia Therefore, after obtaining informed consent from the patient, we started oral administration of steroids at a dose of 20 mg, every 24 h. Thereafter, IgG and IgG4 concentrations decreased rapidly, and the creatinine concentration measured by the enzymatic method, which is routinely performed in our hospital, also improved. The patient’s clinical course is shown in Fig. 3. We are now searching for the cause of the rapid postoperative increase in IgG4, while reducing the steroid dosage.

## Discussion and conclusions

There are two methods for measuring creatinine concentrations: the Jaffe method, which measures the active methylene group, and the enzymatic method using the Trinder reaction. The latter method is the primary method because of its sensitivity and simplicity and is the method used by many medical institutions and laboratories [1]. Our hospital also uses the enzymatic method. However, drugs and abnormal serum proteins can cause errors in the creatinine measurement results [4]. Hummel et al. [5], and Storsley et al. [6] reported that monoclonal IgM causes hypercreatininemia.

All reports of hypercreatininemia indicate IgM as a cause and not IgA or IgG [2, 3]. There are only three known cases of IgG paraproteins causing false lowering of serum creatinine values using the Jaffe method [7]. Our case is considered the first report of pseudohypercreatininemia caused by polyclonal IgG.

Hummel et al. [5] diagnosed pseudohypercreatininemia by high-performance liquid chromatography and the Jaffe method. Although high-performance liquid chromatography could not be performed immediately at our hospital, the presence of pseudohypercreatininemia was quickly inferred from the cystatin C concentration, and combined with inulin clearance, we confirmed normal renal function by measuring creatinine using a differentenzymatic method.

The final diagnosis was made using high-performance liquid chromatography.

Shigunasuoto and Detamina-L are similar enzymatic methods that differ by the buffer solution used. When the creatinine measurement was reproduced manually using Shigunasuoto, white turbidity appeared when the first reagent, the buffer solution, was mixed with the reagent. This turbidity might have increased the absorbance, resulting in a false high creatinine value. This phenomenon did not occur with Detamina-L. Generally, the M protein may become cloudy depending on the pH of the reagent and the concentration of the buffer solution, and this can occur in any enzymatic method.

Reagents used in the Jaffe method may react with sugars, ketones, and cephalosporins in addition to serum creatinine. Both the Jaffe method and the enzymatic method may cause pseudohypercreatininemia; therefore, it is important to confirm the creatinine test method when hypercreatininemia is present without a typical clinical disease course. Our hospital uses the Shigunasuoto enzymatic method because, its positive aspects, including accuracy, compatibility, and cost. Accuracy is limited in any enzymatic method because of potential interference with the M protein.

The strength of this case report is that although it is an event that could occur in any hospital, pseudohypercreatininemia caused by polyclonal IgG has not been reported before; therefore, this is the first report of its kind. A limitation is that there are approximately 30 competing reagents for measuring creatinine. We used Shigunasuoto and Detamina L, which have the largest market share; however, we were unable to validate our findings using other reagents. In addition, the pathological findings of the aorta in this case did not allow for a definitive diagnosis of IgG4-related inflammatory abdominal aneurysm or IgG4-related periarteritis. It is difficult to confirm whether IgG4 is associated with recurrent aortic dissection in this case, and further accumulation of cases is desired.In conclusion, commonly used enzymatic methods of creatinine measurement can lead to pseudohypercreatininemia. We think that pseudohypercreatininemia should be suspected when hypercreatininemia is present in the absence of common renal insufficiency symptoms, such as hyperkalemia, azotemia, and oliguria. When this disease is suspected, we consider it necessary to perform tests to determine the presence of drugs or abnormal proteins that may have caused this disease. We found it useful to measure cystatin C and inulin clearance, as well as useing an enzymatic assay kit to diagnose pseudohypercreatininemia.

## Data Availability

Records and data pertaining to this case are contained in the patient’s secure medical records at Saga University Hospital. If necessary, relevant materials can be provided by the lead author, Ayako Tasaki.

## Abbreviation

IgG: Immunoglobulin G
IgG4: Immunoglobulin G4
IgM: Immunoglobulin M
